# Supplementary material for: Elastocapillary sequential fluid capture in hummingbird-inspired grooved sheets
Source: Nat Commun. 2025 May 27;16:4913. doi: 10.1038/s41467-025-60203-8 (PMC12117096; doi:10.1038/s41467-025-60203-8)
Supplement: Supplementary file 1 — Supplementary Information [file 41467_2025_60203_MOESM1_ESM.pdf]

Supplementary Information for
Elastocapillary sequential fluid capture in hummingbird-inspired grooved sheets

Emmanuel Siéfert
Université libre de Bruxelles (ULB), Nonlinear Physical Chemistry Unit, CP231, 1050 Bruxelles, Belgium and Université libre de Bruxelles (ULB), Nonlinear Physical Chemistry Unit, CP231, 1050 Bruxelles, Belgium

Benoit Scheid and Jean Cappello
Université libre de Bruxelles (ULB), TIPS, 1050, Bruxelles, Belgium

Fabian Brau
Université libre de Bruxelles (ULB), Nonlinear Physical Chemistry Unit, CP231, 1050 Bruxelles, Belgium
(Dated: April 22, 2025)

I. VARYING THE NUMBER OF GROOVES.

Supplementary Figure 5 shows pictures of sheets with identical groove geometry but different number of grooves n.

In the article, we chose to focus on the situation where the width of the system (or equivalently the number of grooves) is adapted in order to perfectly close into a tube (see Supplementary Fig. 5 second row). Hence, when the height of the ribs h and the width of the grooves d is changed, we adjust the number of grooves $n = 2\pi h/d$ (i.e. the width of the system $n(d+w)$) to ensure that it closes in a tube of outer radius $R_{\text{out}} = h(1 + w/d)$ when self contact between adjacent ribs occurs.
If the number of grooves is smaller than n, the ribbon cannot close into a tube and rolls (if sufficiently soft) into a portion of a tube of outer radius $R_{\text{out}} = h(1 + w/d)$ with each groove closed (see Supplementary Fig. 5 first row). The theory for groove closure developed in the article still holds in this case, the only difference is related to the second capillary rise, that does not occur.
When the number of grooves is larger than n, different scenarii can be observed. For a number of grooves slightly larger than $2\pi h/d$ the sheet close into a tube of radius $R_{\text{out}} = (nd + (n+1)w)/(2\pi)$ larger than $h(1 + w/d)$ (see Supplementary Fig. 5 third row), with each groove remaining open. In that case, the theory describing the first capillary rise holds until the closing angle reaches the value $\beta = (n+d)\pi/(nd + (n+1)w)$, for which the structure stops deforming. The dynamics of the second capillary rise taking place in the core of the newly formed tube is more intricate than the one described in the article as the hydraulic resistance is changed because of the increase of the inner radius and the change of boundary condition at the liquid-liquid interface. The gravitational force remains similar to what is derived in the previous section and reads $\mathbf{F}_g = -\pi(R_{\text{out}} - h)^2 \ell_{II} \rho g \mathbf{e}_z$ and the puling capillary force reads $\mathbf{F}_\gamma = \gamma[n(d - 2h\beta) + (n+1)w]\mathbf{e}_z$. Hence the equilibrium height of the second capillary rise is :

$$\ell_{II} = \frac{l_c^2[n(d - 2h\beta) + w(n+1)]}{\pi(R_{\text{out}} - h)^2}. \quad (1)$$

When further increasing the number of grooves, self-contact occurs at the ribbon scale before the groove closes, causing the structures to deform into a more complex, spiral-like shape (see the fourth row of Supplementary Fig. 5). In this scenario, the final height and dynamics are highly dependent on the number of ribs, and deriving the dynamics is quite intricate, as it would require knowledge of the exact shape of the structure. This falls outside the scope of this article.

II. DISCUSSION ON INERTIAL EFFECTS.

In all our calculations we completely disregarded inertial terms. However, at early stages inertia impacts the flow dynamics and leads to an extra term in the fluid momentum equation. The expression of such a force scales as

$$F_{\text{in}} \sim \rho A \ell_{I,II} \frac{d^2 \ell_{I,II}}{dt^2}, \quad (2)$$

with $\ell_{I,II}$ being either the first or the second capillary rise height. The inertial term has to be compared to the viscous force, that, when $d \sim w \sim h \sim R_{\text{int}}$, as in the case in our experiments, scales as

$$F_\mu \sim (\mu A^2/d^4) \ell_{I,II} \frac{d\ell_{I,II}}{dt}. \quad (3)$$

Balancing the two forces, F_{in} and F_μ, we obtain the typical timescale $\tau_{\text{in}} = \rho d^2/\mu$ below which inertia plays a significant role and above which it can be disregarded compared to viscous forces. Experimentally, we used structure of typical dimension $d \sim 700$ µm, fluid density $\rho = 1000$ kg/m^3 and of viscosity equal or larger than $\mu = 10$ mPa s. For such values, $\tau_{\text{in}} \sim 5 \times 10^{-2}$ s.

III. DERIVATION OF THE CAPTURED VOLUME FROM IMAGE ANALYSIS.

When the device is slightly immersed in a liquid, the volume of liquid captured by the initial capillary rise can be determined using the average equilibrium rise height, ℓ_I, and the mean closing angle, β. The first parameter, ℓ_I, can be directly obtained from the raw images. The determination of the second parameter, β, is less straightforward, yet, it can be derived from the measurement of the apparent width of the deformed

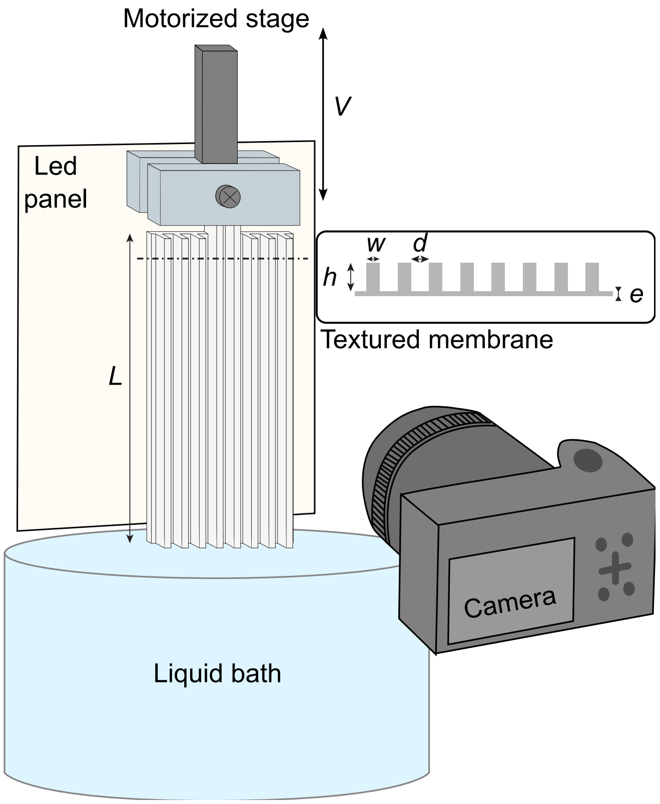

Supplementary Figure 1. **Schematics of the experimental setup.** A grooved sheet hanging from a motorised stage is dip in a wetting liquid bath. The dynamics of the capillary rise is monitored using a camera with a LED panel backlight.

structure Δ (see Fig. 2a of the article). A direct relation exists between β and Δ, expressed as:

$$\Delta = \frac{d+w}{\beta}\sin(n\beta), \quad \text{for} \quad \beta \leq \frac{\pi}{2n} \tag{4}$$

$$\Delta = \frac{d+w}{\beta}, \quad \text{for} \quad \frac{\pi}{2n} \leq \beta \leq \pi. \tag{5}$$

Once the two parameters are known, the volume of liquid captured by the first capillary rise is derived from the equation :

$$\mathcal{V}_I = nA(\beta, \bar{d})\ell_I = nh^2(\bar{d} - \beta)\ell_I. \tag{6}$$

If the structure is not entirely close, no second capillary rise occurs and the total volume of liquid capture is $\mathcal{V} = \mathcal{V}_I$. Yet, if the structure closes, $\beta = \bar{d}/2$, and a second capillary rise of height ℓ_{II} takes place in the core of the newly formed tube of radius $R_{\text{int}} = w/\bar{d}$. In this situation, the total volume of liquid that is captured is $\mathcal{V} = nhd/2\ell_I + \pi(w/\bar{d})^2\ell_{II}$.

Hence, when the structure closes, the measurements of the mean height of the first capillary rise ℓ_I and of the second capillary rise height ℓ_{II} enable the measurement of the total volume of captured liquid.

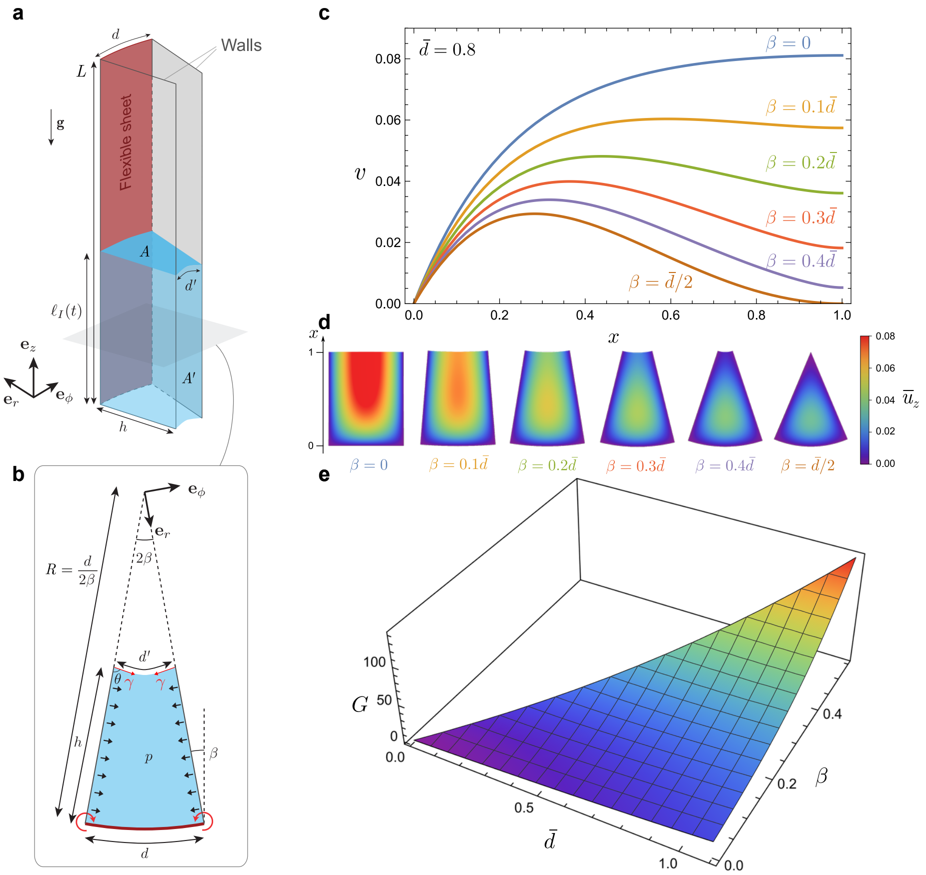

Supplementary Figure 2. **Modelling.** **a**, Capillary rise with a contact angle $\theta = \pi/2$ in an open groove of depth h, width d and making a closing angle β. **b**, Schematic of the mechanical equilibrium: the capillary rise induces a torque through the walls (considered as rigid) due to the negative pressure and the surface tension pulling at the triple line, leading to the bending of the bottom sheet with a radius of curvature $R = d/(2\beta)$. **c**, Function v (Eq. (24b)), representing the dimensionless mean vertical velocity v_z as a function of $x = (R - r)/h$ for $\bar{d} = 0.8$ and various values of $\beta = \sqrt{3}/k$. **d**, Colourmaps of the dimensionless vertical velocity $\bar{u}_z = u_z\,\mu\ell_I/[(\Delta p - \rho g \ell_I)d^2]$ inside the grooves, given by Eqs. (24) and (25), for various values of β and $\bar{d} = 0.8$. **e,** Plot of the dimensionless function G, defined by Eqs. (28) and (29), as a function of the aspect ratio $\bar{d} = d/h$ and the closing angle β of the groove. The colour code reflects the G value.

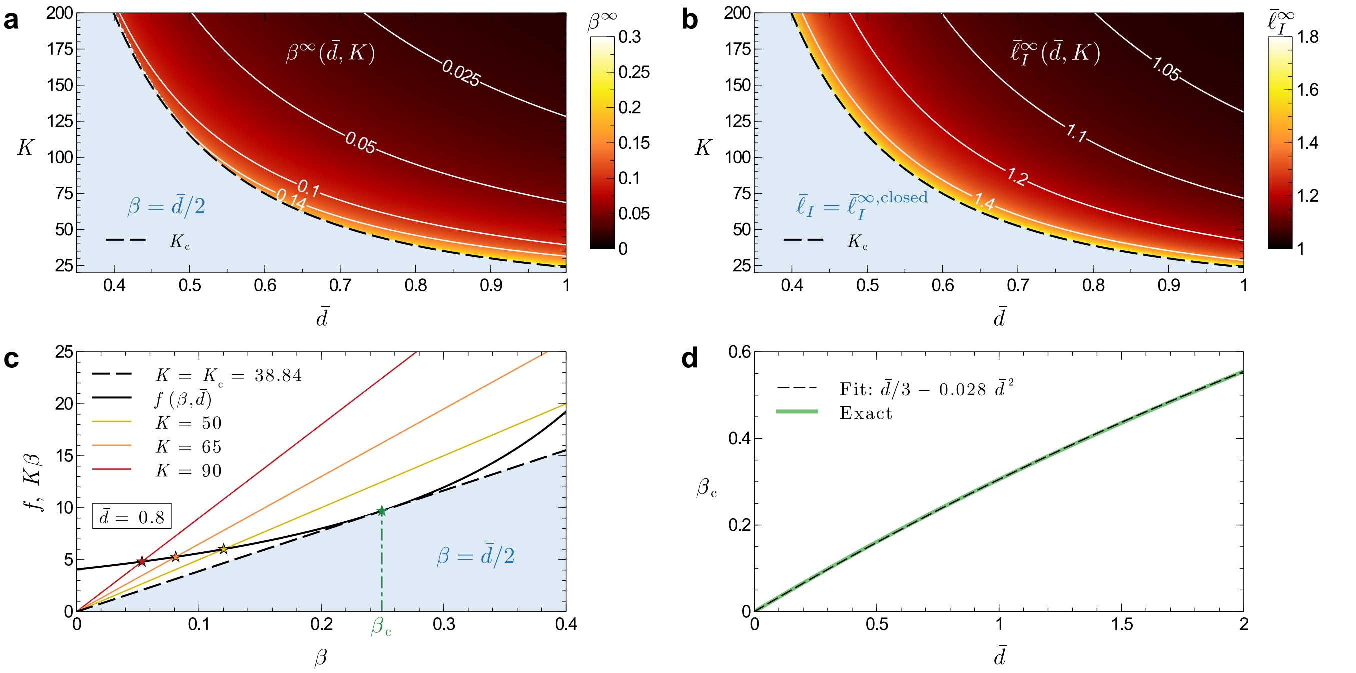

Supplementary Figure 3. Evolution of the equilibrium value of the closing angle, β^∞ defined by Eq. (47), (**a**) and of the liquid height in a groove, $\bar{\ell}_I^\infty$ (**b**), as a function of $\bar{d}$ and K. The critical value $K = K_c$, given by Eq. (52), below which the groove is closed is also shown. **c.** Plots of the right-hand side of Eq. (47) (black solid line) together with the linear elastic relation on the left-hand side as a function of β ($\bar{d} = 0.8$), for various values of the dimensionless stiffness K. Intersection between the two curves, highlighted by stars, yields the static closing angle β at equilibrium. Below a critical value K_c, given by Eq. (52), of the dimensionless stiffness, no solutions exist, and the groove closes until self-contact. **d.** Evolution of β_c, given by Eq. (51), as a function of $\bar{d}$ together with a polynomial approximation.

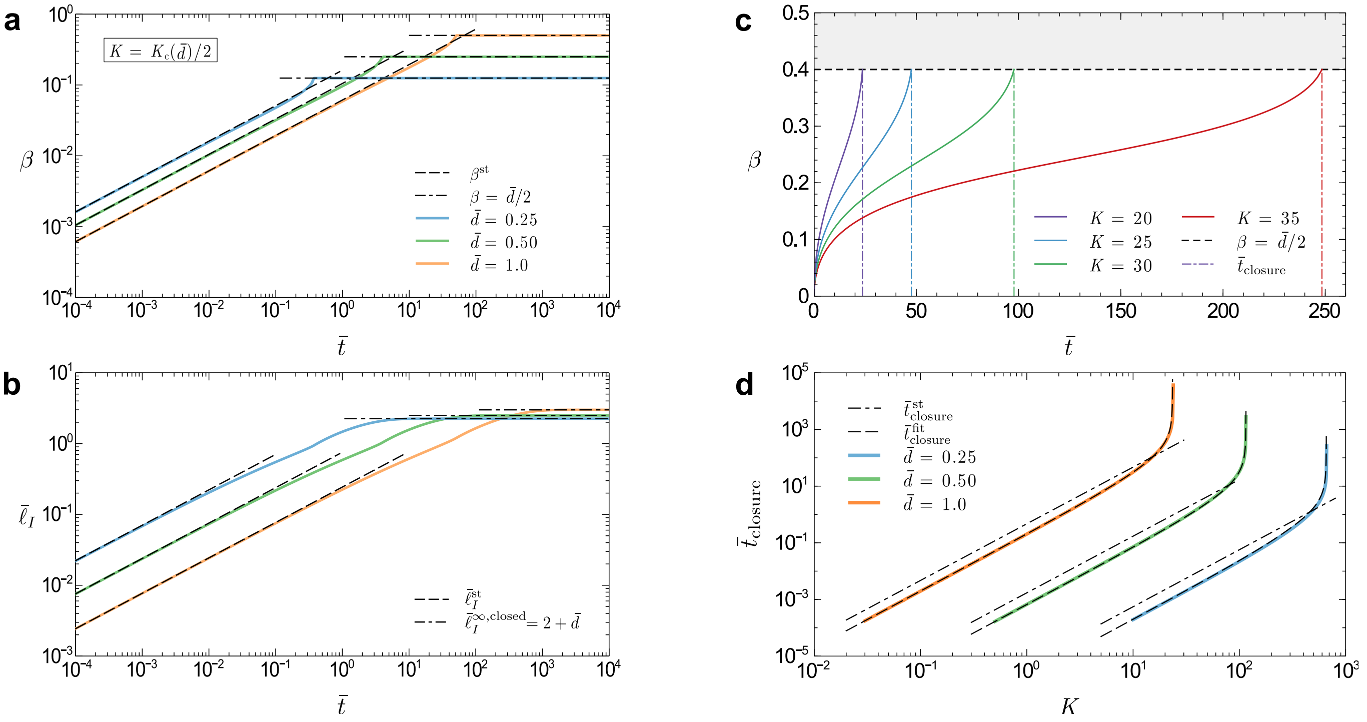

Supplementary Figure 4. Comparison between the solutions for β (**a**) and $\bar{\ell}_I$ (**b**) obtained by solving numerically Eqs. (44), with $K = K_c(\bar{d})/2$ and several values of $\bar{d}$ as indicated, and its asymptotic behaviour at short time (β^{st} and $\bar{\ell}_I^{\text{st}}$, see Eqs. (57) and (58)) and the stationary solution at long time. **c.** Evolution of β as a function of time for $\bar{d} = 0.8$ and several value of $K < K_c = 38.84$. The closure times at which $\beta = \bar{d}/2$ are shown. **d.** Evolution of the closure time, obtained from Eq. (62), as a function of K for several values of $\bar{d}$ as indicated. The estimation (63) of the closure time obtained from the short-time dynamics is shown with dashed-dotted curves and the corrected expression (64) is displayed with dashed curves.

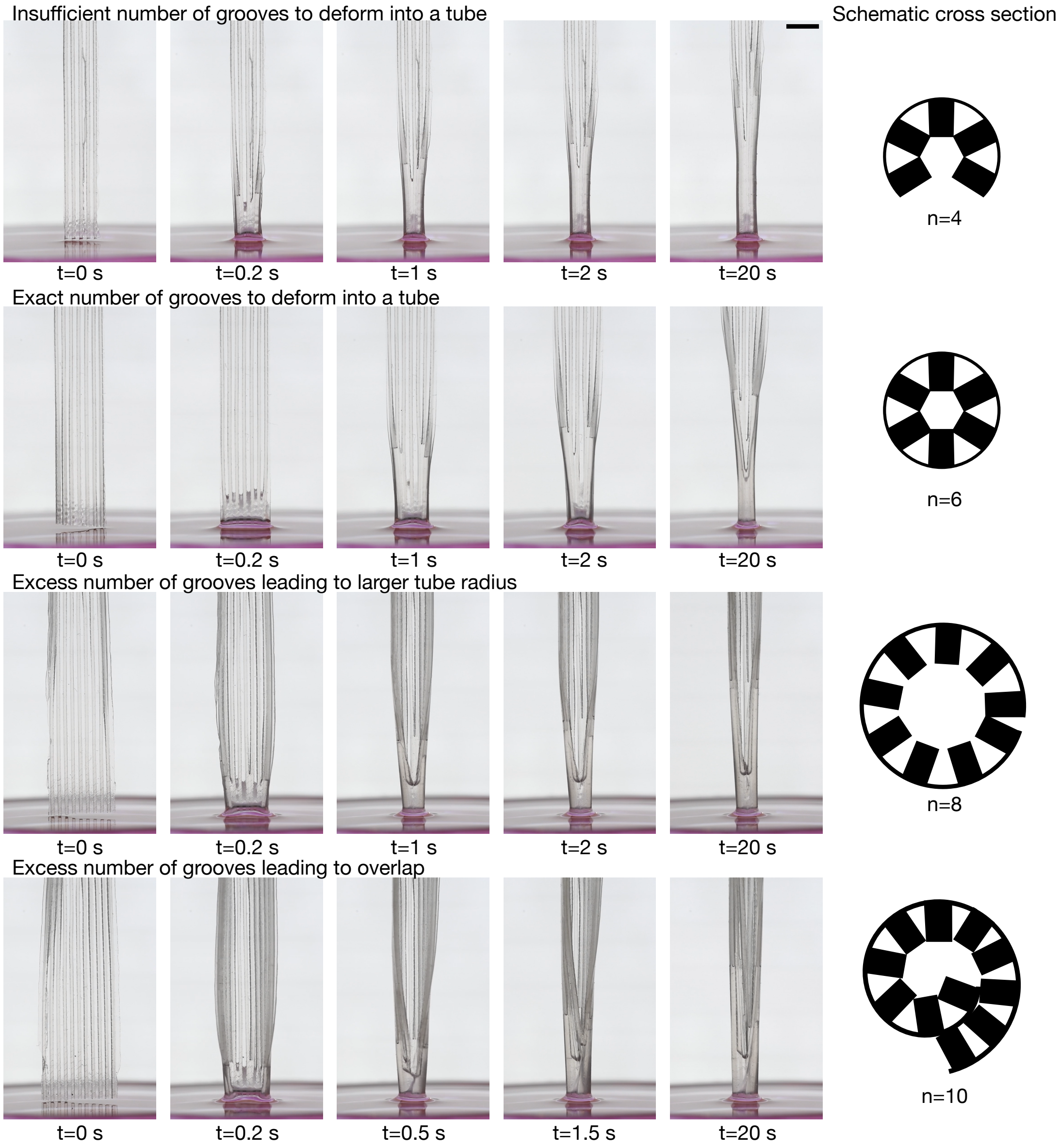

Supplementary Figure 5. **Variation of the Number of grooves**. In the first row, the number of grooves, n, is insufficient to close the sheet into a tube, resulting in the formation of a circular arc. The second row corresponds to the case studied in this article, where the sheet deforms into a tube with an outer radius of $R_{\text{out}} = h(1 + w/d)$. In the third row, an increase in the number of grooves, n, causes the structure to deform into a tube with a outer radius larger than $h(1 + w/d)$, where the top edges of all the ribs do not come into contact. Finally, the fourth row depicts a situation where the structure deforms into a spiral due to overlap.
