# Supplementary material for: Elastocapillary sequential fluid capture in hummingbird-inspired grooved sheets
Source: Nat Commun. 2025 May 27;16:4913. doi: 10.1038/s41467-025-60203-8 (PMC12117096; doi:10.1038/s41467-025-60203-8)
Supplement: Supplementary file 2 — Description of Additional Supplementary Files [file 41467_2025_60203_MOESM2_ESM.docx]

**Supplementary Movie 1:**

Sequential elastocapillary rise.

A ribbed sheet is put in contact with a liquid bath (silicon oil V5). A first capillary rise occurs inside the grooves, inducing a capillary torque on the sheet and the closing of each groove. This results in the global bending of the ribbon into a tubular shape. A second capillary rise takes then place in the newly formed tube. (h=560 µm, w=300 µm, d=250 µm, e=150 µm, L=33 mm).

**Supplementary Movie 2:**

Fluid-structure interaction model.

Side-by-side comparison between an experiment (e=135±10 µm, h=800±50 µm, w=400±40 µm, d=700±40 µm, L=26.1±0.2 mm) and our model without any fitting parameter (for e=129 µm, h=800 µm, w=430 µm, d=670 µm, L=26 mm). Liquid used is silicone oil V10.

**Supplementary Movie 3:**

Sheet thickness controls the closure time.

As they are dipped into the bath, thinner sheets close much faster than thicker ones. Above a critical thickness ($\Lambda>1$), grooves are too stiff to close. Videos have been slowed down 4 times (h=800±50 µm, w=400±40 µm, d=700±40 µm, L=25±1 mm).

**Supplementary Movie 4:** Closure time as a linear viscosity probe.

Two devices of identical geometry (w = 300 ± 30 μm, d = 500 ± 30 μm, h = 600 ± 40 μm, e = 110 ± 10 μm, L = 25.4 μm) are slightly dipped in silicone oil of 20 cSt (left) and 50 cSt (right). As the closure time increases linearly with the viscosity, its measurement could rapidly inform on the liquid viscosity. This could be, for instance, of great help for an indication of the hematocrit level in blood samples which causes a variation in viscosity of several cSt.

**Supplementary Movie 5:**

Flexibility enhances fluid capture during dipping.

Side-by-side comparison between a rigid closed and a flexible open structure (cross sections are shown in insets) as they are dipped into and withdrawn from a bath of silicone oil V1000 at constant speed V=200 mm/min.

The flexible structure, that remains open during dipping, closes during the withdrawing phase, capturing liquid over its whole length, whereas the liquid does not have time to fully penetrate the rigid closed structure.

**Supplementary Movie 6:**

Dipping structures of different stiffnesses.

When the ribbon is too stiff ($\Lambda<1$) it does not curl when withdrawn from the bath, thus capturing a small amount liquid. Above a critical threshold however ($\Lambda>1$), the structure self-assembles into a tubular shape and captures more liquid. Liquid used is ethanol.

**Supplementary Movie 7:**

Device Reopening.

Following closure, the device can be readily reopened via manual mechanical actuation. Residual liquid within the grooves can be effectively removed by swiping the surface.

**Supplementary Movie 8:**

Wall collapse.

When the bottom sheet is thick but the walls sufficiently slender, the capillary rise in the grooves leads to an instability and the collapse of the walls in pairs, promoting a higher capillary rise. Liquid used is silicone oil V10. h=2080 µm, t=1800 µm, w=450 µm and d=680 µm.

**Supplementary Movie 9:**

Surface treatment for the capture of aqueous solutions.

After plasma treatment, the device becomes hydrophilic and can be used to capture aqueous solutions. As the amount of captured liquid depends on the size of the device, by scaling down the device, it may be used to capture liquids available in very small quantities (fraction of blood droplets for instance).

**Supplementary Movie 10:**

Blood capture.

Plasma treatment of the silicone membrane followed by coating with a 2 wt% serum albumin solution enables effective blood capture by the device.

**Supplementary Movie 11:**

Proof of concept of double capillary rise based point-of-care tests.

By functionalizing the grooves of the device with specific markers – here yellow, red, and blue dyes – parallel tests can be achieved in each groove when a liquid imbibes the device. The grooved geometry of the device enables the concomitant fluid capture and aliquoting. The initially open state of the device permits fast liquid capture, while the final closed state forbids contamination and offers a large surface-to-volume ratio that increases the test reliability. After interaction of the liquid with the specific reagents, reading of the test results may be achieved by putting in contact the imbibed device with an absorbent substrate on which a radial spreading takes place and visualization of the colours is eased.
